# Hemorrhage from the Extraction of a Tooth

**Published:** 1842-06

**Authors:** 


*Hemorrhage from the Extraction of a Tooth.*—Dr. Mantell states that in a case of profuse hemorrhage from the extraction of a loose tooth, in a child seven years of age, after all other methods had failed, and a fatal termination was expected, constant pressure was effected by a gold plate accurately adapted to the gum, by a dentist, and the bleeding was effectually suppressed. The means specified by Mr. Roberts, in the 22d No. of the Lancet, had previously been had recourse to with but temporary effect. A paste of plaster of Paris placed on the gum in a soft state, afforded upon its consolidation a firm compression, and the hemorrhage was controlled for several hours; but on taking food the application was loosened, and the bleeding returned. The young lady, the subject of these remarks, had been suffering from purpura hemorrhagica; and on a previous occasion alarming hemorrhage had followed the accidental removal of a loose tooth.—*London Lancet, from Boston Medical and Surgical Journal.*

